# Supplementary material for: An open-source framework for large-scale, flexible evaluation of biomedical text mining systems
Source: J Biomed Discov Collab. 2008 Jan 29;3:1. doi: 10.1186/1747-5333-3-1 (PMC2276192; doi:10.1186/1747-5333-3-1)
Supplement: Additional file 1 — Evaluation platform source code. The additional file contains the software evaluation framework discussed in this paper. The most current release can be downloaded from SourceForge.net [1]. See the accompanying README file for instructions on installation and use. Also included in the distribution is a collection of UIMA wrappers for some commonly used BioNLP tools and annotated corpora. [file 1747-5333-3-1-S1.zip › bionlp_uima_repository-1.0.1/docs/api/src-html/edu/uchsc/ccp/uima/mention/CCPSlotMention_Type.html]

```
001    
002    /* First created by JCasGen Tue Aug 28 16:44:10 MDT 2007 */
003    package edu.uchsc.ccp.uima.mention;
004    
005    import org.apache.uima.cas.FeatureStructure;
006    import org.apache.uima.cas.Type;
007    import org.apache.uima.cas.impl.CASImpl;
008    import org.apache.uima.cas.impl.FSGenerator;
009    import org.apache.uima.cas.impl.TypeImpl;
010    import org.apache.uima.jcas.JCas;
011    import org.apache.uima.jcas.JCasRegistry;
012    
013    /** The superclass for all slot mentions (complex and non-complex).
014     * Updated by JCasGen Tue Aug 28 16:44:10 MDT 2007
015     * @generated */
016    public class CCPSlotMention_Type extends CCPMention_Type {
017      /** @generated */
018      protected FSGenerator getFSGenerator() {return fsGenerator;}
019      /** @generated */
020      private final FSGenerator fsGenerator = 
021        new FSGenerator() {
022          public FeatureStructure createFS(int addr, CASImpl cas) {
023                             if (CCPSlotMention_Type.this.useExistingInstance) {
024                               // Return eq fs instance if already created
025                         FeatureStructure fs = CCPSlotMention_Type.this.jcas.getJfsFromCaddr(addr);
026                         if (null == fs) {
027                           fs = new CCPSlotMention(addr, CCPSlotMention_Type.this);
028                               CCPSlotMention_Type.this.jcas.putJfsFromCaddr(addr, fs);
029                               return fs;
030                         }
031                         return fs;
032            } else return new CCPSlotMention(addr, CCPSlotMention_Type.this);
033              }
034        };
035      /** @generated */
036      public final static int typeIndexID = CCPSlotMention.typeIndexID;
037      /** @generated 
038         @modifiable */
039      public final static boolean featOkTst = JCasRegistry.getFeatOkTst("edu.uchsc.ccp.uima.mention.CCPSlotMention");
040    
041    
042    
043      /** initialize variables to correspond with Cas Type and Features
044            * @generated */
045      public CCPSlotMention_Type(JCas jcas, Type casType) {
046        super(jcas, casType);
047        casImpl.getFSClassRegistry().addGeneratorForType((TypeImpl)this.casType, getFSGenerator());
048    
049      }
050    }
051    
052    
053    
054
```
